# MultiSEss: Automatic Sleep Staging Model Based on SE Attention Mechanism and State Space Model

**DOI:** 10.3390/biomimetics10050288

**Published:** 2025-05-03

**Authors:** Zhentao Huang, Yuyao Yang, Zhiyuan Wang, Yuan Li, Zuowen Chen, Yahong Ma, Shanwen Zhang

**Affiliations:** 1 Xi’an Key Laboratory of High Precision Industrial Intelligent Vision Measurement Technology, School of Electronic Information, Xijing University, Xi’an 710123, China; huangzhentao168@163.com (Z.H.); 15031152189@163.com (Y.Y.); 15716560035@163.com (Z.W.); 17696749100@163.com (Z.C.); 2 School of Physics and Electronic-Electrical Engineering, ABA Teachers College, Wenchuan 623002, China; aba_ly@abtu.edu.cn; 3 School of Electronic Information, Xijing University, Xi’an 710123, China

**Keywords:** sleep staging, multi-scale convolution, SE attention mechanism, state–space model

## Abstract

Sleep occupies about one-third of human life and is crucial for health, but traditional sleep staging relies on experts manually performing polysomnography (PSG), a process that is time-consuming, labor-intensive, and susceptible to subjective differences between evaluators. With the development of deep learning technologies, particularly the application of convolutional neural networks and recurrent neural networks, significant progress has been made in automatic sleep staging. However, existing methods still face challenges in feature extraction and cross-modal data fusion. This paper introduces an innovative deep learning architecture, MultiSEss, aimed at solving key issues in automatic sleep stage classification. The MultiSEss architecture utilizes a multi-scale convolution module to capture signal features from different frequency bands and incorporates a Squeeze-and-Excitation attention mechanism to enhance the learning of channel feature weights. Furthermore, the architecture discards complex attention mechanisms or encoder–decoder structures in favor of a state–space sequence coupling module, which more accurately captures and integrates correlations between multi-modal data. Experiments show that MultiSEss achieved accuracy results of 83.84% and 82.30% in five-fold cross-subject testing on the Sleep-EDF-20 and Sleep-EDF-78 datasets. MultiSEss demonstrates its potential in improving sleep stage accuracy, which is significant for enhancing the diagnosis and treatment of sleep disorders.

## 1. Introduction

Sleep accounts for about one-third of a person’s life and is a fundamental component of human health, influencing aspects such as memory consolidation, emotional regulation, physical recovery, and immune function. Chronic sleep disorders can lead to serious consequences, including cardiovascular diseases [1], diabetes [2], obesity [3], and mental health issues [4]. Sleep plays a crucial role in maintaining overall human health, making it essential to understand and monitor sleep quality and stages.

In clinical settings, the determination of sleep stages typically relies on doctors or specialists performing polysomnography (PSG), which includes electroencephalogram (EEG), electrooculogram (EOG), electromyogram (EMG), and electrocardiogram (ECG).

The frequency of EEG typically ranges from 0.5 Hz to 100 Hz. EOG measures the potential changes around the eyes caused by eye movements, and it is not strictly defined within a fixed frequency range. However, it can capture the relatively low-frequency fluctuations caused by eye movement, typically between 0.1 Hz and 10 Hz, depending on the speed and direction of the eye movement. The frequency range of EMG is generally between 5 Hz and 500 Hz, but in clinical practice, the focus is more often on the range from 5 Hz to 150 Hz. Traditionally, sleep experts or doctors first segment the nighttime PSG data into 30 s non-overlapping segments, and then manually classify these segments into different sleep stages according to sleep manuals (such as Rechtschaffen and Kales (R&K) [5] or American Academy of Sleep Medicine (AASM) standards [6]). For example, according to AASM, sleep time is divided into five stages: wakefulness (W), non-rapid eye movement stage 1 (N1), non-rapid eye movement stage 2 (N2), non-rapid eye movement stage 3 (N3), and rapid eye movement (REM). However, the classification of sleep stages is manually performed by trained technicians through the analysis of PSG data [7]. This process is time-consuming, labor-intensive, and prone to inconsistency due to inter-evaluator variability [8]. Therefore, developing automated methods for sleep stage classification has become especially urgent [9,10,11,12].

Recent studies have shown that deep learning algorithms have achieved significant success in automatic sleep staging. To accomplish this, researchers have widely adopted technologies such as Convolutional Neural Networks (CNNs) and Recurrent Neural Networks (RNNs). Among them, the single epoch model approach has attracted particular attention. This method uses a 30 s unit (i.e., epoch) as the time frame, treating the data of each epoch as input, and constructs deep learning models using CNNs to solve time series classification problems. For example, Xia et al. [13] proposed an end-to-end automatic sleep staging classification method based on raw single-channel sleep EEG signals. This method uses Convolutional Neural Networks (CNN) to extract the time-frequency domain features of the signal, incorporates compression and excitation modules (SE-Block) on the CNN to enhance its feature representation ability, and uses a bidirectional gated recurrent unit (Bi-GRU) to learn the transition rules of sleep stages. Additionally, an attention mechanism is added to the decoding part of the Bi-GRU to strengthen its long-term memory capabilities, highlighting the impact of key features. In order to effectively learn the intrinsic features of significant waves from single-channel EEG signals and capture useful information about sleep stage transition rules, Li et al. [14] proposed a new method, SleepFC. This method includes a Convolutional Feature Pyramid Network (CFPN), Cross-Scale Temporal Context Learning (CSTCL), and a class-adaptive fine-tuning loss function (CAFTLF) based classification network. CFPN learns multi-scale features from prominent waves in EEG signals. CSTCL extracts useful multi-scale transition rules between sleep stages. To address the challenge of effectively extracting temporal information from EEG, which faces issues of future information leakage and high model training time costs, Pan et al. [15] proposed a novel network architecture, CausalAttenNet. It prevents future information leakage through causal dilated convolution and extracts time-related sleep features with a wide temporal receptive field. Traditional recurrent networks are replaced by Multi-Head Attention (MHA), significantly reducing time costs and forming a faster sleep staging network. However, existing methods based on end-to-end CNN or RNN models that implicitly learn temporal correlations may limit the performance of learning time patterns, making accurate phase recognition challenging. Duan et al. [16] proposed a new multimodal and multiscale automatic sleep staging framework, MMS-SleepNet. It uses a deep learning-based multimodal feature extraction module (MMS-FE), incorporating expert knowledge to effectively capture multimodal features for each stage and fine-grained EEG features at various frequencies. The module leverages an attention mechanism to seamlessly integrate the extracted multimodal features, significantly improving classification accuracy. At the same time, a contrastive learning module and data balancing strategy were introduced to address class confusion and data imbalance issues in existing models. Ying et al. [17] proposed a deep learning-based hybrid public–private domain sleep grading network, HybridDomainSleepNet. This network explores the correlation between multimodal data and multiple domains to enhance the representation of different sleep stages. HybridDomainSleepNet can understand both the common and individual characteristics of subjects in different sleep stages and capture long- and short-term dependencies during sleep. Additionally, a multi-domain attention framework was proposed to capture brain signals across domains using a three-branch structure, exploring the correlations of multimodal data across different sleep stages.

There are three main challenges in building an effective sleep stage classification model: first, the difficulty of extracting effective features from raw EEG signals. According to AASM sleep standards, physiological signals in different sleep stages typically exhibit distinct characteristic waveforms, and EEG is time dependent. Therefore, many researchers use spatiotemporal models to extract data features. Secondly, the insufficient capture of cross-modal contextual relationships between different modalities. Different sleep stages are characterized by specific EEG waves, which are also reflected in other signals. Experts typically combine EEG with other signals (such as EOG) to determine sleep stages. For example, EEG waveforms in the REM and N1 stages are similar, while EOG waveforms show significant differences. However, many existing deep learning methods overlook the effectiveness of multimodal data, even though some studies have explored the importance of different modalities [18,19,20]. These studies usually employ simple methods at the model input or intermediate stages, such as concatenation, addition, or dot-product methods, to fuse raw signals or features learned from different modalities together. This leads to the neglect of cross-modal contextual relationships between different modalities, even when they are not in the same epoch. In the end, many model constructions incorporated basic modules such as RNN, LSTM, or Transformer. While they offer better performance, they come with higher computational complexity and costs when processing data like multi-channel multi-lead sleep graphs.

During sleep, the body’s physiological state continuously changes. These changes are reflected not only in individual signals but also in the interactions between multiple signals. For instance, EOG can detect eye movements, which is a fundamental indicator for distinguishing between Rapid Eye Movement (REM) and Slow Eye Movement (SEM). Additionally, the EEG waveforms during the REM and N1 stages are similar, while the EOG waveforms differ significantly. Therefore, when classifying REM and N1 stages, the contribution of EOG is greater than that of EEG. In contrast, the classification of N2 and N3 stages primarily relies on significant waveforms in the EEG. Thus, this paper chose EEG and EOG to construct our model [21].

To address these challenges, MultiSEss was present, which is an innovative deep learning architecture based on convolutional neural networks (CNNs), specifically engineered for automated sleep stage classification. First, this paper designs a multi-scale convolution module to capture low and high-frequency signals across different frequency bands. Next, we implemented a Squeeze-and-Excitation (SE) attention mechanism to obtain channel feature weights. At the end of the model, a state–space sequence coupling module was employed to learn the cross-modal contextual relationships between signals. Compared to traditional one-dimensional convolution or simple weighted fusion methods, the state–space sequence coupling module can more accurately capture correlations between modalities, avoiding information loss caused by modality heterogeneity.

Overall, the contributions of this paper can be summarized as follows: First, multi-scale convolution and SE attention mechanism modules were used to extract frequency features and channel weight features from signals. Second, a state space sequence coupling module was adopted to integrate global context features from multimodal samples and shared features across different samples, enhancing the model’s ability to handle multimodal data. Finally, extensive experiments were conducted on the Sleep-EDF-20 and Sleep-EDF-78 datasets to verify the effectiveness and feasibility of the method.

## 2. Materials and Methods

### 2.1. Sleep-EDF Dataset

In the experiments described in this paper, the Sleep-EDF-20 and Sleep-EDF-78 public datasets obtained from Physiobank [22] were used, as shown in Table 1. Sleep-EDF-20 contains PSG data from two nights for 20 subjects. Sleep-EDF-78 includes PSG data from 78 subjects, serving as an extended version of the Sleep-EDF-20 dataset. Secondly, they wore miniature sleep telemetry systems on the subjects. First, the researchers tested healthy subjects aged between 25 and 101 years, aiming to explore the impact of age on sleep. Secondly, they conducted sleep telemetry. They tested the effects of Temazepam on human sleep without using any other medications. In this dataset, the generated PSG includes EEG data from two channels (Fpz-Cz and Pz-Oz). Both channels have a sampling frequency of 100 Hz. The data for the entire night is recorded in two files: SC*PSG. The hypnograms were manually scored according to the Rechtschaffen and Kales protocol and then labeled as N1, N2, N3, N4, Wake, REM, and UNKNOWN classes. Since stage M and unknown stages do not belong to any sleep stage, they have been excluded from the experiment. In addition, according to AASM standards, stage N3 and stage N4 are combined into a single stage N3 in the experiment. In order to emphasize sleep stages, only the wakefulness within half an hour before and after sleep is included.

### 2.2. The Structure of MultiSEss Model

**Figure 1:**
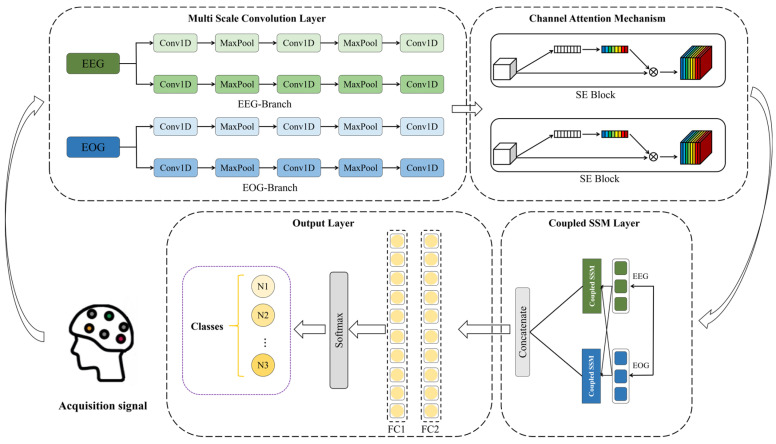
MultiSEss model structure.

Figure 1 illustrates the basic structure of the MultiSEss model. As shown in Figure 1, the MultiSEss model consists of four parts: multi-scale convolutional layers, SE channel attention mechanism layer, SSM coupling layer, and output layer. First, the EEG signals and EOG signals are processed through the multi-scale convolutional layers. The multi-scale convolutional layers capture the features of the signals at various frequencies by using convolution kernels of different sizes, which helps the model understand the complexity of the input data from different dimensions. Each convolution kernel size extracts frequency information within a specific range, ensuring the model’s broad adaptability and sensitivity to the input signals. Subsequently, the features after convolution at different scales are concatenated for each signal. Next, the data passes through the SE attention mechanism, which uses a two-layer neural network to learn the importance of each channel in the feature map from the channel dimension data. This is then multiplied by the original channel data. This module can automatically identify which channel information is more crucial for the final task and adjust the weights of each channel accordingly. Next, the data are passed through the SSM coupling layer to more accurately capture and fuse the correlations between multimodal data. Finally, the classification is output through a two-layer fully connected network and the softmax function.

#### 2.2.1. Multiscale Convolution

**Figure 2:**
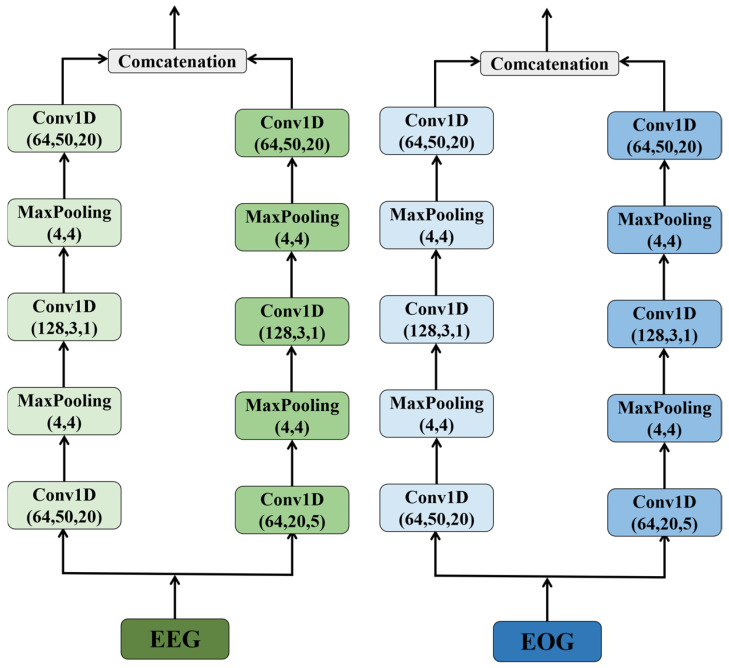
Multiscale convolution module.

In order to extract a more robust temporal representation from the raw sleep signals, this paper introduces a multi-scale convolutional neural network (MSCNN) [23,24]. The MSCNN module is shown in Figure 2 with its core mechanism lying in its feature extraction approach, which utilizes two convolutional layers with parallel filters of different sizes. Specifically, this method uses small-sized kernels to capture low-frequency information in the signal, while large-sized kernels are responsible for capturing high-frequency information. This design allows the model to consider different frequency components simultaneously when processing complex signals, thereby improving recognition accuracy. After each convolutional layer, a max-pooling layer follows, helping highlight the key features within each filter’s scanning region. The max-pooling operation not only simplifies the model’s learning process but also helps the model focus on the most important information points while disregarding less relevant details. For example, in the specific implementation of MSCNN, the large-scale convolutional kernel in the first layer is set to a size of (1, 50), with a stride of 20 and 64 filters; correspondingly, the small-scale convolutional kernel is set to a size of (1, 20), with a stride of 5, and also contains 64 filters. The subsequent max-pooling layer operates with a kernel size of (4, 4). Furthermore, signals from EEG (electroencephalogram) and EOG (electrooculogram) are combined through multiple convolutions and pooling, followed by a Concatenation operation. This step aims to merge two different types of physiological signals in order to generate more comprehensive and representative feature representations for subsequent analysis tasks. This combination not only enhances the model’s understanding of the input signals but also provides strong support for accurate recognition. Overall, MSCNN offers an effective method for processing complex sleep signal data, demonstrating its potential in the medical and health field, especially in sleep research.

#### 2.2.2. Squeeze-and-Excitation Networks

**Figure 3:**
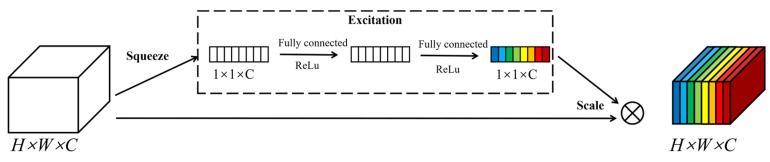
SE attention mechanism module.

The Squeeze-and-Excitation Networks (SENets), first proposed by Hu et al. [25], can scale channel feature maps by explicitly focusing on the interdependence between channels. Since its introduction, the SE module has been widely adopted due to its excellent performance in emphasizing key features and suppressing unnecessary ones. This structure significantly enhances the neural network’s ability to represent features, thereby improving the model’s recognition accuracy. The SE attention mechanism structure is shown in Figure 3.

The working principle of the SE module can be divided into two main stages: Squeeze and Excitation. First, as shown in Equation (1), during the squeezing stage, through the global average pooling operation, the spatial information of each channel in the input feature map U is compressed into a scalar value, forming a C-dimensional vector z, where *C* represents the number of channels in the feature map, *H* is the height, and *W* is the width. Specifically, the global average pooling result z_c_ for each channel C is obtained by averaging all spatial locations of that channel, calculated as

(1) $$z_{c}=\frac{1}{H\times W}\sum_{i=1}^{H}\sum_{j=1}^{W}U_{i,j,c}\;for\;c=1,2,\ldots ,C$$

Here, z_c_ represents the global average pooling result of the C channel.

Subsequently, as shown in formula (2), during the incentive phase, an adaptive weight generation mechanism calculates a corresponding weight value for each channel to achieve selective enhancement or suppression of channels. This process involves two fully connected layers (FC) and nonlinear activation functions (such as ReLU and Sigmoid) to establish relationships between channels. First, the first fully connected layer reduces the dimensionality, transforming the channel descriptor vector *z* into a lower-dimensional space, where *r* serves as the dimensionality reduction ratio. Next, after processing through the ReLU and Sigmoid activation functions, the second fully connected layer maps the low-dimensional representation back to the original dimensions, generating weights for each channel.

(2) $$u=\sigma (W_{2}\delta (W_{1}z))$$

In Equation (2), *δ* represents the ReLU activation function, *σ* represents the Sigmoid activation function, and *W*1 and *W*_2_ are the weighted matrices of the two fully connected layers.

In the final stage of feature recalibration, as shown in Equation (3), the SE module multiplies each channel in the original feature map by its corresponding weight to achieve adaptive channel weighting. The output feature map is calculated as follows:

(3) $$X_{i,j,c}=s_{c}\cdot U_{i,j,c},\;for\;c=1,2,\ldots ,C$$

#### 2.2.3. State Space Model Coupling Module

Currently, in the field of deep learning, although Transformer models are widely popular for their excellent ability to handle long-range dependencies, their enormous parameter size and high computational complexity have become significant bottlenecks. In recent years, the State Space Model (SSM), as an emerging efficient architecture, has shown great potential in handling continuous long-sequence data, and SSM is the core module of Mamba [26,27]. Compared to traditional transformers, SSM offers notable advantages, especially in terms of computational efficiency. The linear time complexity of SSM makes it far more efficient in processing long sequences than the quadratic complexity of transformers, making it particularly suitable for applications that require analysis of ultra-long contexts. Additionally, the mathematical framework of SSM, based on state transition equations and observation equations, provides the model with stronger interpretability. SSM is not limited to natural language processing tasks but can also be applied to time series prediction, speech processing, reinforcement learning, and other fields, demonstrating its powerful capability in solving complex problems. However, SSM has limitations in capturing interaction information between different modalities. To address this issue, this paper proposes a novel module that combines the SSM concept with the attention mechanism—the Multimodal Coupling Module (SSM Coupling Module)—to enhance the interaction performance between modalities. Below is the data processing flow for this module:

The SSM coupling module process is as follows. First, the modal input is defined as a three-dimensional tensor *XnϵR*^B×T×D^, where B represents the batch size, T is the time step, and D is the input feature dimension. The core of the model lies in its effective processing and integration of different modal data. The model includes several key matrices to implement the transformation process from input to output. For example, the state update matrix *AϵR*^H×H^ is responsible for the transformation between hidden states, where H denotes the hidden state dimension. The input projection matrix *BϵR*^H×D^ is used to map the input features to the hidden state space. The modal correlation matrix *S_n_ϵR*^H×H^ aims to capture the relationships between different modalities, thereby enhancing the model’s ability to understand multimodal data. The output transformation matrix is the key in mapping the hidden state back to the original feature space. Additionally, to dynamically evaluate and fuse information from different modalities, the weighted fusion parameter *W_n_* is introduced. This mechanism allows the model to adaptively adjust the contribution of each modality based on the importance of the current input, thereby improving overall performance and flexibility. This design is especially suitable for applications that require handling and integrating various types of data.

State update mechanism: First, based on Equation (4), layer normalization is performed on the input of the *n*-th modality at time step *t* to standardize the input features. Then, according to Equation (5), a cross-modal interaction representation is constructed by aggregating the hidden states of all modalities.

(4) $${\tilde{x}}_{n}(t)=L\mathrm{ayer}N\mathrm{orm}(x_{n}(t))$$

(5) $$H_{sum}(t)=\sum_{n=1}^{N}h_{n}(t-1)$$

Next, as shown in Equation (6), the current input features combined with the historical hidden states of each modality are used to generate the hidden state for the next time step.

(6) $$h_{n}(t)=\tanh(\sum_{m=1}^{M}h_{m}(t-1)\cdot S_{n}+\tilde{X(t)\cdot B^{T}})\in R^{B\times H}$$

Among them, *M* represents the number of modalities, *h_m_*(*t* − 1) is the hidden state of the *m*-th modality at time step *t* − 1, Sn is the correlation matrix of modality *n*, and *B^T^* is the transpose of the input projection matrix.

Cross-modal Interaction and Attention Mechanism:

In order to further enhance the interaction effect between modalities, by combining the historical states of all modalities with the current input, according to Formula (7), the similarity score(*h_n_*(*t*),*h_m_*(*t*)) between modality *n* and modality *m* at time step *t* is calculated to achieve adaptive weighting between modalities. Finally, as shown in Formula (8), the hidden state of modality *n* at time step *t* will interact with the states of other modalities through attention weighting:

(7) $$\alpha_{nm}(t)=\frac{\exp(score(h_{n}(t),h_{m}(t)))}{\sum_{m=1}^{M}\exp(score(h_{n}(t),h_{m}(t)))}$$

(8) $$h_{n}(t)=\tanh(\sum_{m=1}^{M}\alpha_{nm}(t)\cdot h_{n}(t-1)\cdot S_{n}+\tilde{X(t)\cdot B^{T}})$$

Output:

According to Formula (9), the output Yn for each modality is obtained by projecting the corresponding hidden state *h_n_*(*t*) into the output space through the linear transformation matrix *E^T^*. Then, as per Formula (10), the model performs a weighted integration of the outputs from all modalities to generate the final multimodal output *Y*. Here, *W_n_* is the weighting parameter for each modality, which can adaptively adjust the contribution of each modality to the final result.

(9) $$Y=h_{n}\cdot E^{T}$$

(10) $$Y=\frac{1}{M}\sum_{n=1}^{M}W_{n}\cdot Y_{n}$$

### 2.3. Evaluation Indexes

The performance of the proposed model is comprehensively evaluated by several evaluation indexes. Accuracy (ACC) measures the proportion of correct predictions made by the model, but it may not be fully representative in imbalanced datasets. The Macro-F1 score (MF1) takes both precision and recall into account by averaging the F1 scores of each category, providing a holistic evaluation that is not affected by sample distribution, making it particularly suitable for multi-class problems. Cohen’s Kappa (Kappa) measures the consistency between the model’s predictions and the true labels while considering the probability of random agreement, with values closer to 1 indicating higher consistency. Sensitivity (Sen), also known as Recall rate, focuses on the proportion of actual positive samples correctly identified by the model; Specificity (Spec) focuses on the proportion of actual negative samples correctly identified as negative by the model. Precision (Pre) reflects the proportion of samples predicted as positive by the model that are actually positive, showing the accuracy of the model’s predictions. In order to analyze the performance of the model in more detail, two calculation methods are used in this paper. Overall Results provide a comprehensive view of the model’s performance across the entire test set, offering an initial understanding of the model’s overall effectiveness. While Per-Class Precisions, starting from the individual categories, calculate the model’s performance in each category separately, allowing for a detailed display of the differences between categories, which is especially important when dealing with imbalanced class distributions. By combining these two methods, we can assess the model’s actual performance more comprehensively and accurately, ensuring that not only the general trends are observed, but also potential specific issues are identified.

The formulas for the evaluation metrics Accuracy, Macro-F1 score, Cohen’s Kappa, Sensitivity, Specificity, and Precision are shown in (11)–(15). True Positive (*TP*) refers to the model correctly predicting samples that are actually of the positive class as positive. False Negative (*FN*) refers to the model incorrectly predicting samples that are actually of the positive class as negative. False Positive (*FP*) refers to the model incorrectly predicting samples that are actually of the negative class as positive. True Negative (*TN*) refers to the model correctly predicting samples that are actually of the negative class as negative. *p_o_* is the observed agreement probability, and *p_e_* is the expected agreement probability.

(11) $$accuracy=\frac{TP+TN}{TP+TN+FP+FN}$$

(12) $$Macro-F1\;score=\frac{2}{\frac{1}{precision}+\frac{1}{recall}}$$

(13) $$Cohen^{’}Kappa=\frac{p_{o}-p_{e}}{1-p_{e}}$$

(14) $$S\mathrm{ensitivity}=\frac{TP}{TP+FN}$$

(15) $$Specificity=\frac{TN}{TN+FP}$$

(16) $$precision=\frac{TP}{TP+FP}$$

## 3. Experimental Results and Analysis

### 3.1. Experimental Setup

In the experiment, this paper used a five-fold cross-validation method across subjects to evaluate model performance on two datasets. Specifically, participants in each dataset were randomly and evenly divided into five groups, ensuring that each fold accurately reflected the overall sample’s feature distribution. This method not only enhanced the reliability of the results but also effectively reduced the impact of individual differences on model evaluation, maximizing data utilization and evaluation effectiveness. During the model training phase, the Adam optimizer with a learning rate of 0.001 was chosen. The Adam optimizer combines the advantages of AdaGrad and RMSProp, providing an adaptive learning rate that dynamically adjusts based on each parameter’s historical gradient, making it especially suitable for handling sparse gradients. To address the class imbalance issue in the dataset, a weighted cross-entropy loss function was employed, with the importance weights of different class samples being adaptively adjusted to ensure balanced information acquisition from all categories by the model during the learning process. Additionally, to ensure consistency in the data used for both the training and testing sets, all data from the pre-trained model was set with the same random seed, shuffled randomly, and then sent to the network model. To further enhance the model’s expressiveness and generalization ability, the batch size was configured as 128 throughout the training process, with 100 training epochs being systematically executed. This configuration aims to optimize through extensive iterations, enabling the model to learn effectively from diverse data, thereby improving its predictive ability and stability. These strategies work together to ensure the reliability of the experimental results and the model’s high performance.

### 3.2. Result of MultiSEss Model

This study focuses on evaluating the performance of a novel MultiSEss model in sleep signal recognition from EEG data. The effectiveness of the MultiSEss model was comprehensively validated through comparative experiments with several state-of-the-art deep learning algorithms. The models involved in the comparison include U-time [28], ResnetLSTM [29], Cross-Modal Transformer [30], AttnSleep [31], and MMASleepNet [32]. The experimental results, as shown in Table 2 and Table 3, reveal that among the six models evaluated, MMASleepNet performs the worst. Specifically, on the Sleep-EDF-20 dataset, MMASleepNet’s Accuracy, Macro-F1 score, Cohen’s Kappa coefficient, sensitivity, specificity, and Precision are 79.04%, 71.87%, 70.02%, 78.33%, 94.81%, and 70.55%, respectively; In the Sleep-EDF-78 dataset, these metrics are 76.62%, 69.53%, 67.92%, 78.05%, 94.26%, and 67.39%. In comparison, the proposed MultiSEss model demonstrates superior performance. On the Sleep-EDF-20 dataset, the model achieved evaluation metrics of 83.84%, 73.46%, 75.36%, 72.20%, 95.31%, and 95.15%; for the Sleep-EDF-78 dataset, these values were 82.30%, 72.23%, 74.21%, 71.10%, 95.02%, and 74.63%.

In addition, considering model complexity, U-time and ResnetLSTM have higher computational demands due to the use of recursive or convolutional neural network structures, especially when processing long time series. Cross-Modal Transformer has a higher computational cost because of its complex attention mechanism. While AttnSleep uses attention mechanisms to enhance feature extraction capabilities, its design also results in a relatively higher number of parameters and computational load. In contrast, although MMASleepNet has lower performance, it shows certain advantages in terms of computational resource consumption, which may be related to its relatively simple architecture. However, it is worth noting that the proposed MultiSEss model, despite including several modules (such as MSCNN, SE attention mechanism module, and SSM module), ensures high performance while maintaining a reasonable level of computational complexity and parameter count through careful design. This allows it to not only provide high-precision sleep staging results in practical applications but also effectively control the use of computational resources.

Furthermore, the study observed that the classification accuracy for N1, N3, and REM sleep stages was relatively high for both the Sleep-EDF-20 and Sleep-EDF-78 datasets. This indicates that the MultiSEss model proposed in this paper can more effectively perform feature extraction and fusion operations when processing multimodal electrophysiological signals, leading to more accurate sleep staging. This result further demonstrates the advantages of the MultiSEss model over other baseline methods.

### 3.3. Ablation Study

**Figure 4:**
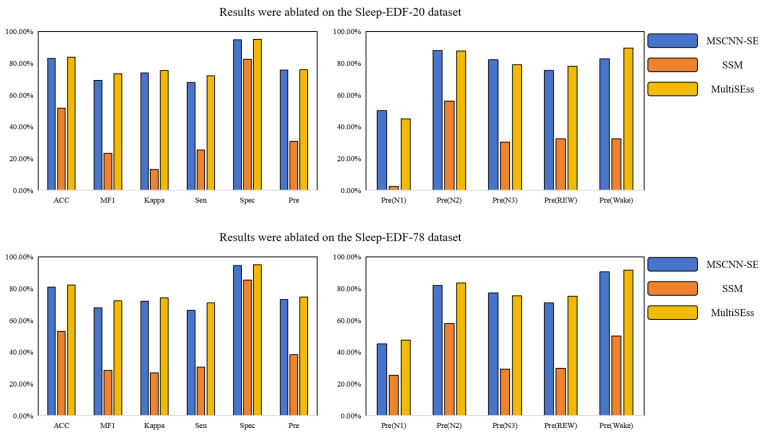
MultiSEss ablated experimental results on two datasets, Sleep-EDF-20 and Sleep-EDF-78.

To further explore the contributions of the spatial module and temporal module in the MultiSEss model, ablation tests were carried out on the Sleep-EDF-20 and Sleep-EDF-78 datasets, using a five-fold cross-subject experimental setup. The results of the ablation experiments are presented in Table 4 and Table 5, as well as Figure 4. Specifically, MultiSEss is composed of the MSCNN module and the SE attention mechanism module. and the SSM module. The MSCNN and SE attention mechanism modules collaborate to extract spatial features, while the SSM module is dedicated to capturing temporal features. The individual importance of these two modules can be better understood through systematic comparison of their effects on the overall model performance. Based on the data in Table 4 and Table 5, from the Sleep-EDF-20 dataset, the spatial feature extraction model that only includes the MSCNN and SE attention mechanism (MSCNN-SE) achieved an accuracy of 82.99%, while the complete MultiSEss model, integrating all three modules, achieved an accuracy of 83.84%. Similarly, on the Sleep-EDF-78 dataset, MSCNN-SE achieved an accuracy of 81.01%, while the complete MultiSEss model reached an accuracy of 82.30%. From these results, it can be seen that, although the MSCNN-SE combined model’s performance is slightly lower than that of the entire MultiSEss model, the performance of the individual SSM module is significantly lower. This suggests that the spatial features extracted by the MSCNN-SE module dominate the overall model performance. However, it is noteworthy that, when the SSM module is added to enhance the model’s ability to capture temporal features, the overall performance of the model is further enhanced. This finding highlights the significance of modeling the interdependence between features, particularly temporal features. In addition to focusing on spatial features when dealing with complex physiological signal data, this study demonstrates that, while spatial feature extraction is a key factor in improving classification performance, combining temporal feature modeling can further optimize the model’s performance. This is crucial for achieving more accurate sleep stage recognition.

## 4. Conclusions

This paper presents a multi-channel sleep signal automatic classification model, MultiSEss, based on EEG and EOG signals. With its unique design, the model efficiently extracts key features from complex EEG signals and uses these features to achieve precise sleep stage classification. MultiSEss employs a multi-scale convolutional neural network to extract rich feature information from EEG data across different frequency bands. Additionally, it incorporates a channel attention mechanism module to identify and weight the data features from each channel, thereby enhancing the model’s focus on important features. Furthermore, to ensure that the global context information of each signal is effectively captured, MultiSEss introduces an SSM module to further improve classification accuracy. In experimental evaluation, MultiSEss was tested on two widely used datasets, Sleep-EDF-20 and Sleep-EDF-78, with five-fold cross-validation accuracy reaching 83.84% and 82.30%, respectively, proving the model’s superior performance. It is worth noting that MultiSEss is an end-to-end model network that can directly learn features from raw sleep signals and perform classification without relying on manually crafted feature extraction processes. This not only improves work efficiency but also demonstrates the model’s potential in future development of efficient and accurate real-time brain–computer interface frameworks. The dataset will be systematically expanded in both scale and diversity in future work to enable more comprehensive evaluation of the model’s performance. To enhance the model’s robustness in noisy data environments, a specialized masking module is being developed to effectively mitigate noise interference.

Subsequent optimization efforts will prioritize performance enhancement of the MultiSEss model, encompassing systematic exploration of its integration within sleep signal-based usability testing frameworks. These refinements are projected to not only strengthen the model’s robustness and prediction accuracy but also significantly expand its practical deployment scenarios.

## Figures and Tables

**Table 1:** Sample size and sample proportion in the SLEEP-EDF dataset.

Dataset	W	N1	N2	N3	REM	Total
Sleep-EDF-20	8285 19.6%	2804 6.6%	17,799 42.1%	5703 13.5%	7717 18.2%	42,308
Sleep-EDF-78	65,951 14.3%	21,522 3.2%	69,132 43.7%	13,039 18.5%	25,835 20.3%	195,479

**Table 2:** Performance of automatic installment on the Sleep-EDF-20 dataset.

Model	Overall Results (%)						Per-Class Precisions (%)				
	ACC	MF1	Kappa	Sen	Spec	Pre	Pre(N1)	Pre(N2)	Pre(N3)	Pre(REW)	Pre(Wake)
U-time	80.52	72.07	71.87	77.86	95.03	70.56	37.66	92.99	57.24	75.89	89.02
ResnetLSTM	81.96	73.60	73.50	75.51	95.15	72.73	37.92	90.77	68.87	76.12	89.97
Cross-Modal Transformer	81.29	72.49	72.73	76.12	95.13	70.60	36.76	92.72	61.76	73.82	87.94
AttnSleep	81.33	72.44	72.66	75.59	95.09	70.51	36.02	92.81	64.42	73.53	85.70
MMASleepNet	79.04	71.87	70.02	78.33	94.81	70.55	33.36	94.24	57.16	77.13	90.83
MultiSEss	83.84	73.46	75.36	72.20	95.15	75.87	45.07	87.69	79.01	78.04	89.56

**Table 3:** Performance of automatic installment on the Sleep-EDF-78 dataset.

Model	Overall Results(%)						Per-Class Precisions(%)				
	ACC	MF1	Kappa	Sen	Spec	Pre	Pre(N1)	Pre(N2)	Pre(N3)	Pre(REW)	Pre(Wake)
U-time	79.74	71.84	70.97	73.50	94.79	69.78	38.97	84.42	60.04	72.94	92.53
ResnetLSTM	79.18	71.50	70.87	76.57	94.75	69.12	40.15	88.86	52.08	70.48	94.18
Cross-Modal Transformer	79.16	71.84	70.97	77.82	94.79	69.50	39.26	89.52	50.15	74.63	93.92
AttnSleep	80.06	73.62	72.27	79.49	95.01	70.72	42.00	89.16	53.91	73.25	95.29
MMASleepNet	76.62	69.53	67.92	78.05	94.26	67.39	38.57	88.37	41.09	73.90	95.04
MultiSEss	82.30	72.23	74.21	71.10	95.02	74.63	47.62	83.51	75.36	75.11	91.58

**Table 4:** Ablation experiment results of MultiSEss on the Sleep-EDF-20 dataset.

Model	Overall Results (%)						Per-Class Precisions (%)				
	ACC	MF1	Kappa	Sen	Spec	Pre	Pre(N1)	Pre(N2)	Pre(N3)	Pre(REW)	Pre(Wake)
MSCNN-SE	82.99	69.32	73.93	67.99	94.89	75.70	50.10	87.99	82.13	75.44	82.84
SSM	51.83	23.39	13.23	25.41	82.59	30.80	2.50	56.30	30.34	32.35	32.55
MultiSEss	83.84	73.46	75.36	72.20	95.15	75.87	45.07	87.69	79.01	78.04	89.56

**Table 5:** Ablation Experiment Results of MultiSEss on the Sleep-EDF-78 Dataset.

Model	Overall Results (%)						Per-Class Precisions (%)				
	ACC	MF1	Kappa	Sen	Spec	Pre	Pre(N1)	Pre(N2)	Pre(N3)	Pre(REW)	Pre(Wake)
MSCNN-SE	81.01	67.98	72.17	66.38	94.60	73.20	45.23	81.96	77.30	70.99	90.52
SSM	53.15	28.54	27.02	30.72	85.45	38.51	25.35	58.11	29.21	29.79	50.12
MultiSEss	82.30	72.23	74.21	71.10	95.02	74.63	47.62	83.51	75.36	75.11	91.58

## Data Availability

Restrictions apply to the availability of these data. Data were obtained from [physiobank] and are available from the authors/at Sleep-EDF Database Expanded.
